# Thickened Gallbladder Wall, Abdominal Pain, and Fever: A Rare Presentation of Dengue in Rural Australia

**DOI:** 10.7759/cureus.73684

**Published:** 2024-11-14

**Authors:** Jinghong Zhang, David Dalton

**Affiliations:** 1 General Surgery, Goulburn Valley Health, Shepparton, AUS

**Keywords:** abdominal pain, dengue fever, deranged liver function tests, diagnostic dilemma, thickened gallbladder wall

## Abstract

Dengue is endemic in tropical and subtropical regions, affecting millions of people worldwide. While it is not endemic to Australia, outbreaks can occur in Queensland each year. The rarity of dengue in other regions of Australia may pose a diagnostic challenge when patients present at hospitals. We report a rare case of dengue in Victoria, where the patient arrived at the Emergency Department with abdominal pain, fever, abnormal liver function tests, and a thickened gallbladder wall on imaging. The patient was referred to General Surgery for a possible diagnosis of acute cholecystitis. However, the indication for an emergency cholecystectomy was unclear due to the absence of other radiological features. The diagnostic challenge persisted until the patient tested positive for dengue virus serology.

## Introduction

Dengue virus (DENV) infection is the leading mosquito-borne viral disease globally. It has four serotypes (DENV-1, DENV-2, DENV-3, DENV-4). Infection with any of the serotypes can lead to various clinical outcomes, with the majority of cases (70%-80%) being asymptomatic [1]. However, patients may develop symptoms including fever, nausea, vomiting, joint and muscle pain, and abdominal pain [2]. In the state of Victoria, the incidence of dengue remains low in the regional areas (16% of reported cases between 2010 and 2016), despite an increasing number of infections by 22% per year statewide over the last decade [3]. From 2013 to 2016, half of the patients with dengue infection presented to hospitals. Among these patients, 33% were admitted for management [3]. As a result, timely diagnosis of dengue can be challenging in rural Victoria, especially when patients present with a clinical picture suggestive of an acute abdomen. Here, we report a rare case of dengue in Shepparton, where a 28-year-old patient presented to the Emergency Department with abdominal pain and fever, accompanied by deranged liver function tests and a markedly thickened gallbladder wall. She was admitted to General Surgery and underwent a surgical evaluation to determine the cause of her symptoms.

## Case presentation

A 28-year-old female presented to the Emergency Department at Goulburn Valley Health in regional Victoria with a three-day history of worsening upper abdominal pain. She also complained of intermittent nausea and vomiting, as well as a few episodes of loose bowel movements. She was otherwise healthy and had not undergone any surgery in the past. There was no recent travel history. In addition, she is an immigrant from the Solomon Islands and works at a regional meat processing factory in Victoria.

On examination, she had a soft abdomen but reported tenderness in the right upper quadrant with positive Murphy’s sign. There was no rebound tenderness. She did not appear jaundiced. Her body temperature was 38.7°C at initial presentation, along with a heart rate of 105 beats per minute, systolic blood pressure of 105 mmHg, diastolic blood pressure of 75 mmHg, respiratory rate of 22 breaths per minute, and an oxygen saturation of 97% on room air. In terms of blood test results, her C-reactive protein (CRP) was significantly elevated (Table 1), and she also had deranged liver function test results. Furthermore, she was leukocytopenic and thrombocytopenic.

**Table 1:** Laboratory investigation results CRP, C-reactive protein; AST, aspartate aminotransferase; ALT, alanine aminotransferase; ALP, alkaline phosphatase; GGT, gamma glutamyl transferase

Parameters	Obtained values (day 1 of admission)	Obtained values (day 4 of admission)	Reference range
Hemoglobin	131 g/L	119 g/L	115–155
Hematocrit	0.34 L/L	0.35 L/L	0.33–0.45
White cell count	3.4 x 10^9^/L	7.3 x 10^9^/L	4.0–12.0
Neutrophils	2.7 x 10^9^/L	2.2 x 10^9^/L	2.0–8.0
Lymphocytes	1.1 x 10^9^/L	4.6 x 10^9^/L	1.0–3.5
Eosinophils	0.0 x 10^9^/L	0.0 x 10^9^/L	0.0–0.5
Platelet count	49 x 10^9^/L	147 x 10^9^/L	150–400
Bilirubin	33 umol/L	8 umol/L	1.0–20.0
CRP	185 mg/L	19 mg/L	<5.0
AST	89 U/L	100 U/L	5–30
ALT	109 U/L	128 U/L	5–35
ALP	293 U/L	288 U/L	30–110
GGT	114 U/L	152 U/L	5–35

**Figure 1:**
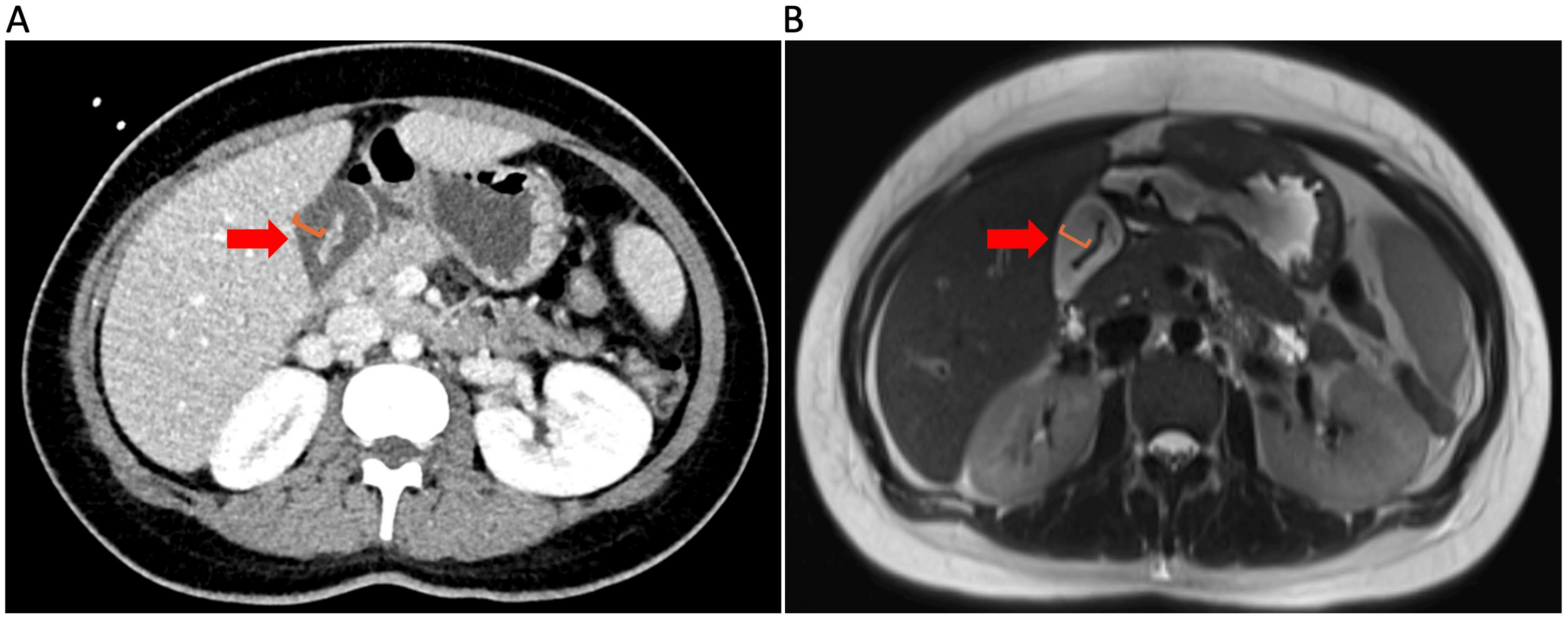
The axial images of a thickened gallbladder on imaging. Arrows in red indicate the gallbladder, which has a thickened wall and a collapsed lumen. Scales in orange show the thickened gallbladder wall. These radiological characteristics were demonstrated on CT (A) and MRCP (B). CT, computed tomography; MRCP, magnetic resonance cholangiopancreatography

The patient was referred to General Surgery for a possible diagnosis of acute cholecystitis. Intravenous antibiotics were commenced. Surprisingly, the abdominal ultrasound showed a thickened contracted gallbladder with no gallstones or sludge. The common bile duct had a diameter of 4.5 mm without any evidence of choledocholithiasis (Figure 1A). There was no abnormality in the liver, pancreas, spleen, or kidneys. These features were consistent with the findings from a subsequent magnetic resonance cholangiopancreatography (MRCP), which revealed a gallbladder wall thickness of 15 mm (Figure 1B). Again, no cholelithiasis or choledocholithiasis was demonstrated. There was no pericholecystic fluid either. Of note, there was a trace of free fluid identified at the inferior aspect of the liver, which suggested possible hepatitis.

Due to the lack of radiological evidence to confirm the diagnosis of acute cholecystitis, an emergency cholecystectomy was not clearly indicated. However, over the next 24 hours of surgical admission, the patient continued to feel unwell and reported ongoing upper abdominal pain. A medical consultation was requested to explore the non-surgical causes of the patient’s presenting complaint. A variety of blood tests, including screening for autoimmune and infectious diseases, were sent off for analysis. On day 3 of admission, the patient had significant clinical improvement, with CRP trending down to 19 mg/L. Most of the blood tests advised by the medical team returned negative, including the hepatitis screen and Q fever. The fecal microscopy, culture, and sensitivity (MCS) test was also negative. On day 4, the patient was evaluated as suitable for discharge. She was booked for an elective interval cholecystectomy.

On day 1 post-discharge, the surgical team was informed by the pathologist that the patient tested positive for dengue fever, with positive IgM and IgG on serology. This was tested using standard Dengue Virus Serology with Enzyme-Linked Immunosorbent Assay (ELISA), which was run by Austin Pathology. The Department of Health was immediately notified. The patient was also contacted with a plan to repeat the serology test in two weeks. The elective cholecystectomy was cancelled, and a medical follow-up was arranged. During the medical follow-up, it was found that the repeat serology test remained positive for IgM and IgG. However, the patient had fully recovered clinically.

## Discussion

Although millions of dengue infections are reported globally each year, the incidence remains low, with a median of 1,466 cases reported annually from 2012 to 2022 in Australia [4]. While most patients with dengue are asymptomatic, some may experience an acute febrile illness that can vary from undifferentiated fever to dengue hemorrhagic fever and shock. Traditionally, dengue can be diagnosed by detecting viral RNA using reverse transcription polymerase chain reaction (RT-PCR) or by assessing the presence of antibodies through serological testing [5]. For symptomatic patients, the management primarily focuses on supportive care with fluids and simple analgesics, as there is no specific antiviral treatment for the virus [6].

Considering the low incidence of dengue and its resemblance to acute abdomen presentations, the emphasis on the work-up for a surgical cause of the symptoms may be excessive. In our case, establishing a clear diagnosis became more challenging when it was found that the patient had a markedly thickened gallbladder wall. Interestingly, gallbladder wall thickening (GBWT) has been reported to be associated with dengue infections, and its degree may indicate the severity of the infection. It is speculated that the pathophysiology of GBWT in dengue is secondary to increased capillary permeability [7].

Acute cholecystitis is one of the differential diagnoses when patients present with right upper quadrant abdominal pain. The Tokyo Guidelines, which incorporate sonographic findings, provide diagnostic criteria for acute cholecystitis [8]. Sonographic features of acute cholecystitis include gallstone, sludge, GBWT, pericholecystic fluid, wall edema, gallbladder distention, and the sonographic Murphy sign [9]. It is important to note that approximately 95% of patients with acute cholecystitis have gallstones [10]. Despite having significant GBWT, the patient in our case was categorized as having “suspected” rather than “definite” acute cholecystitis due to the absence of other radiological features. Although acalculous cholecystitis may be an alternative differential diagnosis, it is rare in adults and often associated with certain conditions such as congestive heart failure, diabetes, systemic vasculitis, and malignancies [11].

Further history was obtained from the patient after the diagnosis. She lived in a shared accommodation with a few friends who had recently returned from the Solomon Islands. Most of her friends experienced mild symptoms such as cough and headache. However, she was the only one who developed severe abdominal pain and fever. Whilst surgical conditions cannot be overlooked when patients present with abdominal pain and elevated inflammatory markers, dengue infection should be considered one of the differential diagnoses, especially in the context of a thickened gallbladder wall without evidence of gallstones. In Australia, 94% of reported dengue cases are imported [4]. It is therefore important to include a detailed travel history and early laboratory diagnosis to provide patients with appropriate management.

## Conclusions

The timely diagnosis of dengue fever can be challenging in Australia due to its rarity, especially when patients present with symptoms that can resemble an acute abdomen, such as fever and abdominal pain. The findings of highly elevated CRP levels and a markedly thickened gallbladder wall may complicate the diagnosis. The implications of this case report are that, while a surgical work-up is urgently needed, suspicion for dengue should be raised in patients with relevant risk factors such as recent travel to endemic areas.
